# Dysphagia Caused by Right-Sided Aortic Arch and Lusorian Artery

**DOI:** 10.5935/abc.20160203

**Published:** 2017-02

**Authors:** Julio Gil, Bruno Marmelo, Davide Moreira, Luís Ferreira dos Santos, José Costa Cabral

**Affiliations:** Serviço de Cardiologia, Centro Hospitalar Tondela Viseu

**Keywords:** Deglutation Disorders / complications, Aorta, Thoracic, Cough, Subclavian Artery Syndrome

**Figure 1:**
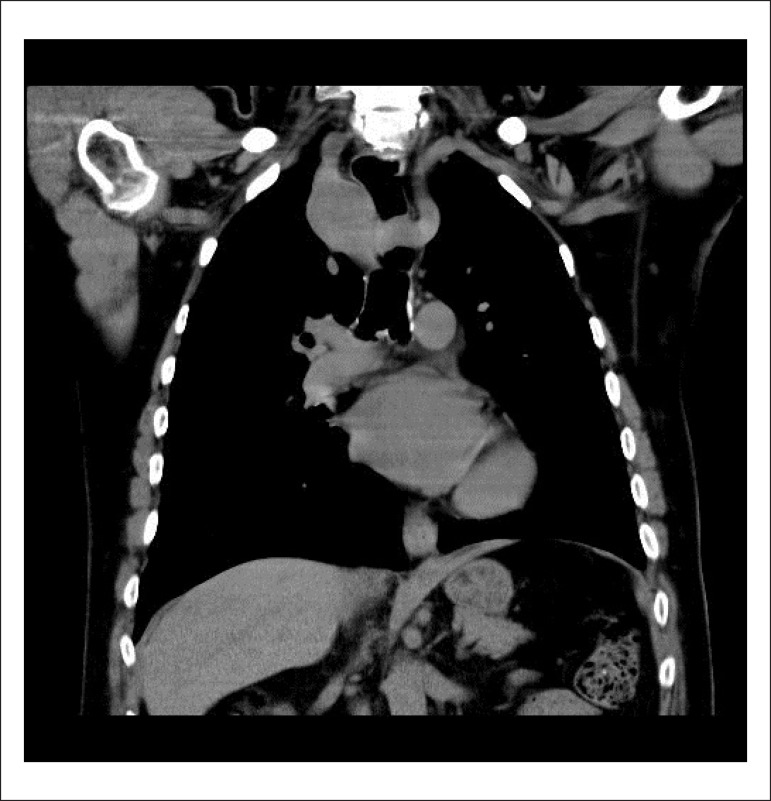
Neuhauser vascular anomaly.

This case report describes a 75-year-old male patient presenting with persistent cough. His chest X-ray showed a right-sided paratracheal opacification. A thoracic CT (Figure 1 and Video 1) was subsequently performed to better characterize the radiographic findings. The exam revealed a Neuhauser Vascular Anomaly, that pertains a right-sided aortic arch and lusorian artery. 

**Video 1:** Watch the videos here: http://www.arquivosonline.com.br/2017/english/10802/video_ing.asp

The lusorian artery is also known as an aberrant right subclavian artery. It is an anatomical variant in which the brachiocephalic artery is absent. Thus, from the aortic arch there are 4 originating arteries: the right and left common carotid arteries and the right and left subclavian arteries. The lusorian artery prevalence varies from 0.16 to 0.8%, depending on the country. The anomaly is clinically asymptomatic in more than 90% of the cases. Most symptoms arise in individuals at advanced ages, probably related to atherosclerotic phenomena. The most frequent symptoms are dysphagia, dyspnea, retro-sternal pain, coughing and weight loss. The lusorian artery and the right-sided aortic arch coexist in 9.2% of the cases.

